# Scalp Seborrheic Dermatitis Severity Is Associated with Lipid and Liver-Associated Biochemical Parameters

**DOI:** 10.3390/jcm15186937

**Published:** 2026-09-08

**Authors:** Marta Pośpiech, Kamila Zawadzinska-Halat, Maciej Pastuszczak

**Affiliations:** Clinical Department of Dermatology, Medical University of Silesia, 40-055 Katowice, Poland; martapospiech94@gmail.com (M.P.); zawadzinskakamila@gmail.com (K.Z.-H.)

**Keywords:** seborrheic dermatitis, scalp, lipid metabolism, triglycerides, gamma-glutamyl transferase

## Abstract

**Introduction**: Seborrheic dermatitis (SD) is a lipid-dependent inflammatory dermatosis in which sebaceous activity, *Malassezia*, barrier dysfunction and host inflammation interact. Whether scalp SD severity is associated with systemic lipid and liver-associated biochemical parameters remains unclear. **Methods**: In this exploratory cross-sectional observational study, we included 34 adults with clinically diagnosed scalp SD. Severity was assessed using a published 16-point scalp SD score and a study-specific 0–36 physician-assessed score. Fasting lipids, glucose, alanine aminotransferase (ALT), aspartate aminotransferase and gamma-glutamyl transferase (GGT) were recorded. The triglyceride/high-density lipoprotein cholesterol ratio and non-HDL cholesterol were calculated. Patients were stratified as having non-severe or severe disease according to the 16-point score. **Results**: Complete 16-point data were available in 33 patients: 15 with non-severe and 18 with severe scalp SD. Severe disease was associated with higher triglycerides (116.0 vs. 78.0 mg/dL; *p* = 0.004), triglyceride/high-density lipoprotein cholesterol ratio (2.57 vs. 1.23; *p* = 0.006), ALT (46.0 vs. 20.0 U/L; *p* = 0.028) and GGT (33.0 vs. 11.0 U/L; *p* = 0.003). The strongest correlations with the 16-point score were observed for GGT (rho = 0.745), triglycerides (rho = 0.634), ALT (rho = 0.572) and triglyceride/high-density lipoprotein cholesterol ratio (rho = 0.569; all *p* < 0.001). These prespecified associations remained significant after false-discovery-rate adjustment and after adjustment for age and BMI. The correlation pattern was strengthened using the 0–36 score and persisted after excluding one liver enzyme outlier. **Conclusions**: More severe scalp SD was associated with triglyceride-related lipid parameters and liver-associated enzymes, suggesting a broader lipid-liver metabolic phenotype. These findings require confirmation in larger controlled studies.

## 1. Introduction

Seborrheic dermatitis (SD) is a chronic, relapsing inflammatory dermatosis with a predilection for sebaceous-rich areas, particularly the scalp, face and upper trunk [1,2]. Scalp involvement is often regarded as a benign or cosmetic problem, yet persistent scaling, erythema and pruritus may be clinically burdensome and associated with impaired quality of life [3]. Despite its high prevalence and everyday relevance in dermatological practice, SD remains a disease whose pathogenesis is only partly understood.

The scalp represents a unique lipid-rich cutaneous microenvironment, where sebaceous gland activity, epidermal barrier function, resident microorganisms and host immune responses interact continuously. The classical pathogenic model of SD is based on three interconnected elements: colonization by *Malassezia* spp., availability of sebaceous lipids and individual host susceptibility [4,5,6]. *Malassezia* yeasts are lipid-dependent organisms capable of hydrolysing sebaceous triglycerides through lipase activity, generating free fatty acids and other lipid-derived metabolites that may promote irritation, inflammation, abnormal desquamation and barrier impairment in susceptible individuals [5,6].

However, SD should no longer be considered a purely yeast-driven disorder. Transcriptomic analyses of dandruff and scalp SD have shown a distinct inflammatory molecular signature, with reciprocal upregulation of immune-response genes and downregulation of genes involved in lipid metabolism [7]. Structural and lipidomic studies have also demonstrated impaired barrier function and alterations in stratum corneum ceramide composition in lesional SD skin [8]. These observations support a broader model in which *Malassezia* acts within a permissive host environment characterized by altered lipid handling, inflammatory activation and defective epidermal barrier integrity [2,4,7,8,9].

The present study focused specifically on scalp SD for several reasons. First, the scalp is one of the most frequently and persistently involved anatomical sites in SD and represents a particularly lipid-rich environment, where sebaceous activity and lipid-dependent microbial metabolism are central to disease expression. Second, scalp disease can be quantified using site-specific clinical severity assessment, allowing analysis of disease activity rather than only the presence or absence of SD. Third, persistent scalp erythema, adherent scaling and pruritus are common reasons for specialist referral and may reflect a more chronic, persistent or diagnostically challenging disease phenotype. Therefore, scalp SD provides a clinically accessible model in which to explore whether local lipid-dependent inflammation is associated with systemic lipid and liver-associated biochemical parameters.

This lipid-centred biology raises an important clinical question: whether SD, especially when involving the scalp, may also reflect systemic metabolic disturbances. The relationship between SD and metabolic health has recently attracted increasing attention. A systematic review on nutrition, obesity and SD highlighted that dietary patterns, obesity, micronutrient abnormalities and metabolic factors may influence SD risk or severity, although available studies remain heterogeneous [10]. In clinical studies, SD has been associated with metabolic syndrome and its components, including dyslipidaemia, increased triglycerides and reduced high-density lipoprotein cholesterol [11,12]. Other studies have reported correlations between serum lipid parameters and sebum secretion, as well as associations between lipid profile abnormalities and SD severity [13,14]. However, these studies generally addressed SD as a broader clinical entity and did not specifically evaluate quantified scalp SD severity in relation to both lipid profile and liver-associated enzyme levels. Taken together, these findings suggest that the “seborrheic” phenotype may not be limited to local skin surface lipids, but may also intersect with systemic lipid metabolism.

The liver is central to lipid and lipoprotein homeostasis, triglyceride handling and metabolic inflammation. In this context, metabolic dysfunction-associated steatotic liver disease (MASLD) is currently defined by hepatic triglyceride accumulation in the presence of cardiometabolic risk factors [15]. Only limited data have addressed liver-related abnormalities in patients with SD. A retrospective study reported hepatic steatosis in a substantial proportion of patients with SD who underwent abdominal ultrasonography, particularly among older patients, men and those with metabolic comorbidities; however, SD severity was not graded, scalp involvement was not specifically quantified, and liver-associated biochemical markers were not correlated with cutaneous disease activity [16]. Thus, whether objectively assessed scalp SD severity is associated with biochemical indicators of lipid and liver-associated metabolic dysfunction remains poorly defined.

Clarifying this relationship may be clinically relevant. If severe scalp SD is associated with dyslipidaemia or liver-associated biochemical abnormalities, SD could represent not only a local inflammatory scalp disorder, but also a visible marker of a broader metabolic phenotype. Therefore, the present exploratory study aimed to evaluate associations between scalp SD severity and selected metabolic and liver-associated parameters, with particular emphasis on triglycerides, the triglyceride/high-density lipoprotein cholesterol ratio, alanine aminotransferase and gamma-glutamyl transferase. We hypothesized that more severe scalp SD would be associated with a more adverse lipid and liver-associated biochemical profile.

## 2. Materials and Methods

### 2.1. Study Design and Patients

This exploratory cross-sectional observational study was conducted at the Department of Dermatology in Zabrze, Medical University of Silesia, between January 2026 and June 2026. The outpatient clinic functions as a regional referral centre for patients with diagnostically or therapeutically challenging dermatological conditions, including scalp disorders. Patients may attend the clinic directly or be referred by dermatologists working in local outpatient practices when further diagnostic assessment or specialist management is required.

Consecutive adult patients presenting for the first time to our centre with clinically diagnosed scalp seborrheic dermatitis during the study period were screened for eligibility. Patients were included if they fulfilled the clinical diagnostic criteria for scalp seborrheic dermatitis (SD) and had available clinical severity assessment and fasting laboratory data.

Scalp SD was diagnosed by the same board-certified dermatologist on the basis of typical clinical morphology and distribution, including scalp erythema, scaling or adherent flaking, and pruritus. This approach is consistent with the widely accepted view that SD is primarily a clinical diagnosis based on lesion appearance and location [1,17]. All severity assessments were performed by the same dermatologist before laboratory results were reviewed.

Patients with clinical features suggesting another primary scalp disorder, including scalp psoriasis, tinea capitis, scarring alopecia or other inflammatory scalp diseases, were not included. In doubtful cases, differential diagnosis was based on clinical assessment and, where appropriate, additional diagnostic procedures.

Patients with autoimmune diseases, except for Hashimoto thyroiditis, were excluded. Additional exclusion criteria included current treatment with systemic immunosuppressive drugs, chronic viral hepatitis B infection, and a history of hepatitis C virus infection.

Demographic and clinical data were recorded, including age, sex, body mass index (BMI), scalp disease severity and pruritus intensity. Lifestyle-related factors potentially relevant to lipid profile and liver-associated biochemical parameters were also collected, including alcohol exposure, cigarette smoking and physical activity. In addition, information on chronic comorbidities and regular medication use was obtained, with particular attention to arterial hypertension, type 2 diabetes, hypercholesterolaemia, hypothyroidism and lipid-lowering therapy. Patients receiving lipid-lowering therapy were not excluded in order to reflect a real-world dermatology referral population; lipid-lowering therapy was recorded and addressed in descriptive comparisons and exploratory sensitivity analyses. These clinical, lifestyle-related and treatment-related variables were considered potential confounders and were addressed, where appropriate, in descriptive analyses, parsimonious partial correlation analyses and exploratory sensitivity analyses. The presence of *Malassezia* spp., if assessed as part of the routine diagnostic work-up, was recorded for exploratory analyses.

Alcohol exposure was recorded from medical history as a categorical self-reported variable: no current alcohol use, occasional alcohol use, documented problematic alcohol use, or unavailable data. Because quantitative data on alcohol intake were not available, alcohol exposure could not be expressed in grams per day and was used only as a descriptive covariate and in sensitivity analyses.

Physical activity was recorded by structured medical history as self-reported usual recreational physical activity during the 3 months preceding clinical assessment. Patients were categorized as having no/low physical activity, occasional physical activity, guideline-consistent physical activity, or unavailable data. Guideline-consistent physical activity was defined as at least 150 min per week of moderate-intensity aerobic activity, at least 75 min per week of vigorous-intensity aerobic activity, or an equivalent combination.

### 2.2. Assessment of Scalp Seborrheic Dermatitis Severity

Disease severity was assessed using two complementary clinical instruments.

The primary severity measure was the 16-point scalp SD scale proposed by Zhang et al. [18]. This scale includes three domains: adherent scalp flaking, scored from 0 to 10 points; maximum erythema area, scored from 0 to 3 points; and pruritus, scored from 0 to 3 points. The total score ranges from 0 to 16 points. According to the proposed classification, a total score of 0–5 points indicates mild disease, 6–9 points moderate disease and 10–16 points severe disease [18]. For group comparisons, patients with a 16-point score below 10 were classified as having non-severe disease, whereas those with a score of 10 or higher were classified as having severe disease.

In addition, a study-specific 0–36 physician-assessed scalp SD severity score was used as a secondary, exploratory and more granular measure of objective scalp disease activity. This score was developed to provide a topographical assessment of inflammatory and scaling changes across the scalp. It was calculated in four scalp quadrants. In each quadrant, erythema was scored from 0 to 4, scaling/flaking from 0 to 4, and Wood’s lamp fluorescence from 0 to 1, giving a maximum of 9 points per quadrant and 36 points in total. Higher scores indicated more severe scalp disease. The full scoring scheme is provided in Supplementary Figure S1. The 0–36 score was assigned during the clinical visit before laboratory results were reviewed; therefore, the assessor was blinded to the biochemical parameters at the time of severity scoring. However, the score has not been externally validated and was not subjected to formal inter-rater or intra-rater reliability testing. For this reason, it was used only as a secondary exploratory measure, whereas the published 16-point scalp SD score remained the primary severity measure.

Pruritus intensity was also recorded separately using a 0–10 numerical rating scale, where 0 indicated no pruritus and 10 indicated the most severe imaginable pruritus.

### 2.3. Laboratory Assessment

Available laboratory parameters were recorded from the initial diagnostic evaluation performed at our centre. Clinical severity assessment and blood sampling were performed on the same day. Venous blood samples were collected in the morning after an overnight fast. Patients were instructed to avoid food intake during the evening and night preceding blood sampling, in accordance with routine fasting blood collection procedures.

The analysis focused on metabolic and liver-associated variables, including serum triglycerides, total cholesterol, high-density lipoprotein cholesterol (HDL-C), low-density lipoprotein cholesterol (LDL-C), glucose, alanine aminotransferase (ALT), aspartate aminotransferase (AST) and gamma-glutamyl transferase (GGT). Laboratory parameters were measured in the hospital central diagnostic laboratory using standardized automated biochemical methods according to local laboratory procedures.

The triglyceride/HDL-C ratio was calculated by dividing triglyceride concentration by HDL-C concentration, using the same units. Non-HDL cholesterol was calculated as total cholesterol minus HDL-C. These derived lipid indices were included because they may better reflect atherogenic and insulin-resistance-associated lipid patterns than isolated lipid parameters.

### 2.4. Study Outcomes and Variables

The prespecified primary biochemical variables of interest were triglycerides, TG/HDL-C ratio, ALT and GGT. These variables were selected a priori because they reflect triglyceride-rich lipid metabolism and liver-associated biochemical profile, which were central to the study hypothesis.

The primary outcome of the study was the association between the 16-point scalp SD severity score and these prespecified biochemical variables. Secondary analyses included between-group comparisons of these parameters in patients with non-severe and severe scalp SD, correlations using the study-specific 0–36 physician-assessed scalp SD severity score, and sensitivity analyses addressing selected potential confounders. Other biochemical parameters, including total cholesterol, HDL-C, LDL-C, non-HDL-C, glucose and AST, were considered exploratory variables.

### 2.5. Sample Size

This exploratory cross-sectional study was based on a fixed available cohort of consecutive eligible first-time patients presenting to our centre during the predefined recruitment period from January 2026 to June 2026. No formal a priori sample size calculation was used to determine recruitment. However, because the planned primary analyses were correlation analyses between the 16-point scalp SD severity score and prespecified biochemical variables, a sample size calculation was performed to estimate whether the available cohort was adequate to detect a clinically meaningful correlation.

Assuming a two-sided significance level of 0.05, 80% statistical power and a minimum meaningful correlation coefficient of rho = 0.50, 29 evaluable patients would be required for a correlation analysis based on Fisher’s z transformation. The available number of patients with complete primary 16-point severity data was 33, and the exact denominators for the prespecified primary correlation analyses ranged from 32 to 33. Therefore, the study had sufficient power to detect moderate-to-large correlations of approximately rho ≥ 0.50, but it was underpowered to reliably detect smaller associations or to support robust multivariable modelling. Consequently, the study should be interpreted as exploratory and hypothesis-generating, and the findings require confirmation in larger controlled cohorts.

### 2.6. Statistical Analysis

Because of the exploratory character of the study and the limited sample size, the analysis was primarily descriptive and hypothesis-generating. Continuous variables are presented as medians and minimum–maximum ranges unless otherwise stated. Categorical variables are presented as numbers and percentages. Missing data were handled by complete-case analysis for each specific comparison or correlation. No imputation was performed because of the exploratory nature of the study and the limited sample size. Exact denominators are reported for variables that were not available in all participants.

Patients were compared according to scalp SD severity defined by the 16-point score. Severe disease was defined as a score of 10 or higher, whereas scores below 10 were classified as non-severe. Between-group comparisons for continuous variables were performed using the Mann–Whitney U test. Binary categorical variables were compared using Fisher’s exact test. Multicategory categorical variables, including alcohol exposure and physical activity categories, were compared using the Fisher–Freeman–Halton exact test. These between-group comparisons were considered descriptive.

Associations between scalp SD severity scores and metabolic or liver-associated parameters were assessed using Spearman’s rank correlation coefficient. The 16-point scalp SD score was used as the primary severity measure. The study-specific 0–36 physician-assessed score was analysed as a secondary severity measure. The prespecified primary biochemical variables were triglycerides, TG/HDL-C ratio, ALT and GGT. These variables were selected because they reflect triglyceride-rich lipid metabolism and liver-associated biochemical profile, which were central to the study hypothesis. Other biochemical parameters, including total cholesterol, HDL-C, LDL-C, non-HDL-C, glucose and AST, were considered exploratory variables. Correlations were visualized using ranked correlation plots.

For correlation analyses, 95% confidence intervals for Spearman’s rho were calculated using Fisher’s z transformation. To address multiple testing in the correlation analyses, false-discovery-rate adjustment was performed using the Benjamini–Hochberg method. False-discovery-rate adjustment was applied separately to the correlation analyses based on the 16-point score and the 0–36 score. Both unadjusted and false-discovery-rate-adjusted *p*-values are reported.

To further address potential confounding while avoiding overfitting, exploratory rank-based partial correlation analyses were performed for the prespecified primary biochemical variables: triglycerides, TG/HDL-C ratio, ALT and GGT. These analyses assessed the associations between the 16-point scalp SD severity score and each biochemical variable after adjustment for age and BMI. Age and BMI were selected because age differed between severity groups and BMI is biologically related to lipid and liver-associated biochemical parameters. Because of the limited sample size, more extensive multivariable models including multiple lifestyle, medication and comorbidity variables were not performed.

Sensitivity analyses were performed to assess the robustness of the main findings. The primary correlation analyses were repeated after exclusion of the patient with extreme liver enzyme elevations. Additional sensitivity analyses were performed after exclusion of patients receiving lipid-lowering therapy, patients with documented problematic alcohol use, current smokers and patients reporting no or low physical activity. These analyses were considered exploratory because of the reduced sample size in each subgroup.

A two-sided *p*-value below 0.05 was considered statistically significant. Because of the exploratory nature of the study and the limited sample size, all *p*-values should be interpreted as descriptive and hypothesis-generating, even when formal statistical significance was reached.

Statistical analyses were performed using Python 3.13.5 with pandas 2.2.3, SciPy 1.17.0 and NumPy 2.3.5. Figures were generated using matplotlib 3.10.8.

### 2.7. Ethical Approval

The study was conducted in accordance with the Declaration of Helsinki and was approved by the Bioethics Committee of the Medical University of Silesia in Katowice, Poland [approval number: BNW/NWN/0052/KB1/127/I/25/26, date: 20 January 2026]. All patients provided written informed consent for participation and use of anonymized clinical and laboratory data.

## 3. Results

### 3.1. Patient Characteristics According to Scalp Seborrheic Dermatitis Severity

Thirty-four patients with scalp seborrheic dermatitis were included in the study. Complete data for the 16-point scalp SD severity score were available in 33 patients, who were included in severity-stratified analyses. Of these, 15 patients were classified as having non-severe scalp SD and 18 as having severe scalp SD.

Clinical, biochemical, lifestyle-related and potentially confounding characteristics according to disease severity are shown in Table 1. Patients with severe scalp SD were younger than those with non-severe disease, with a median age of 27.5 versus 34.0 years, respectively (*p* = 0.017). Sex distribution did not differ significantly between groups. BMI was numerically higher in patients with severe scalp SD, but this difference did not reach statistical significance.

Detection of *Malassezia* was markedly more frequent in patients with severe scalp SD than in those with non-severe disease (16/18, 88.9% vs. 3/15, 20.0%; *p* < 0.001).

The distribution of selected comorbidities and medications potentially affecting lipid or liver-associated biochemical parameters was broadly comparable between severity groups. Lipid-lowering therapy was reported in 1/15 patients with non-severe scalp SD and 3/18 patients with severe scalp SD (*p* = 0.607). Hypertension was present in 5/15 and 6/18 patients, respectively (*p* = 1.000), whereas type 2 diabetes was uncommon and was reported in only one patient with severe disease. Thyroid hormone replacement and urate-lowering therapy were also infrequent and did not differ significantly between groups. Current smoking was more common in patients with severe scalp SD, but the difference was not statistically significant (7/18, 38.9% vs. 3/15, 20.0%; *p* = 0.283). Alcohol exposure categories were similarly distributed between groups (*p* = 1.000). Patients with severe scalp SD more frequently reported no or low physical activity; however, the overall distribution of physical activity categories did not reach statistical significance (*p* = 0.140).

### 3.2. Lipid and Liver-Associated Parameters According to Disease Severity

**Figure 1:**
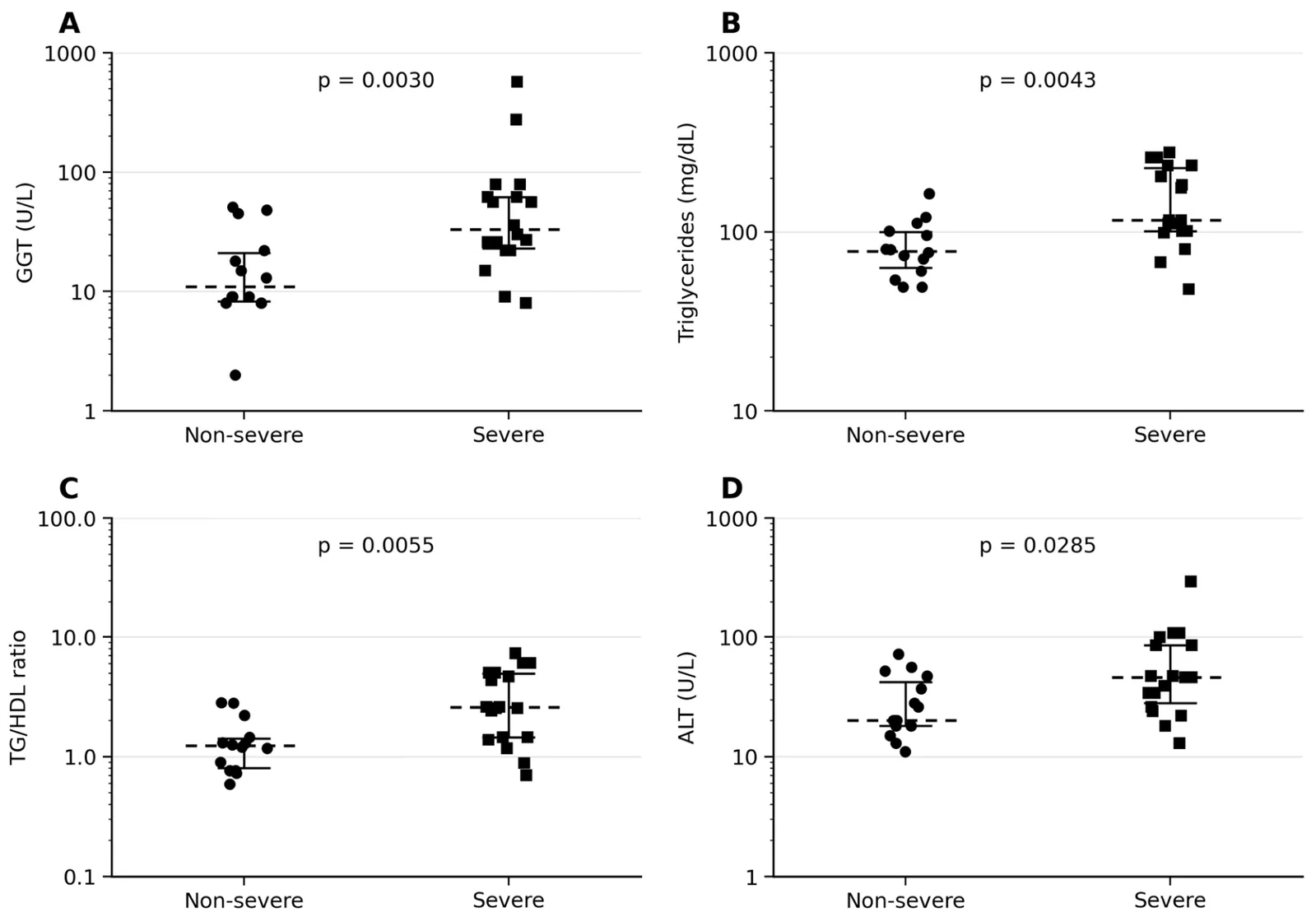
Metabolic and liver-associated parameters according to scalp seborrheic dermatitis severity. Distribution of (**A**) gamma-glutamyl transferase (GGT), (**B**) triglycerides, (**C**) triglyceride/high-density lipoprotein cholesterol ratio (TG/HDL ratio), and (**D**) alanine aminotransferase (ALT) in patients with non-severe and severe scalp seborrheic dermatitis. Disease severity was classified using the 16-point scalp seborrheic dermatitis score; severe disease was defined as a score ≥ 10. Dashed horizontal lines indicate medians, and vertical whiskers indicate the interquartile range. Individual patients with non-severe disease are shown as black circles, whereas those with severe disease are shown as black squares.

Patients with severe scalp SD showed higher values of selected metabolic and liver-associated parameters than patients with non-severe disease (Table 1, Figure 1). Median triglyceride levels were higher in the severe group than in the non-severe group (116.0 vs. 78.0 mg/dL; *p* = 0.004). A similar difference was observed for the TG/HDL-C ratio, which was more than twofold higher in patients with severe disease (2.6 vs. 1.2; *p* = 0.006).

Liver-associated enzymes also differed between severity groups. Median ALT was higher in patients with severe scalp SD than in those with non-severe disease (46.0 vs. 20.0 U/L; *p* = 0.028). The difference was even more pronounced for GGT, with median values of 33.0 U/L in the severe group and 11.0 U/L in the non-severe group (*p* = 0.003). The distribution of these parameters is presented in Figure 1.

### 3.3. Correlations Between Scalp Seborrheic Dermatitis Severity and Metabolic Parameters

**Figure 2:**
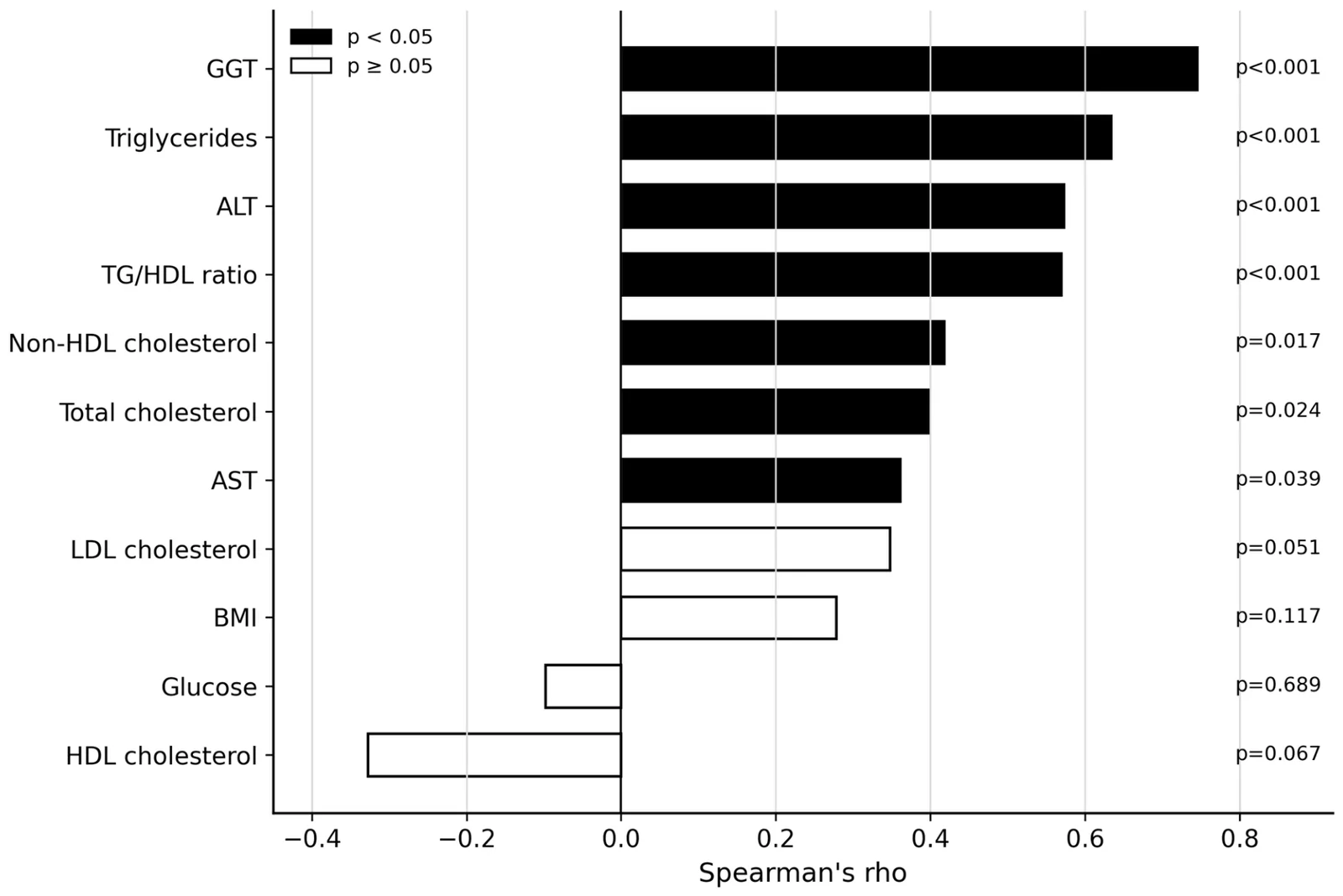
Correlations between scalp seborrheic dermatitis severity and metabolic or liver-associated parameters. Bars represent Spearman’s rank correlation coefficients between the 16-point scalp seborrheic dermatitis severity score and selected biochemical or anthropometric parameters. Filled black bars indicate correlations with unadjusted *p* < 0.05, whereas open bars indicate correlations with unadjusted *p* ≥ 0.05. Reported *p*-values are unadjusted; false-discovery-rate-adjusted *p*-values are provided in Table 2. Positive values indicate increasing parameter values with higher disease severity, whereas negative values indicate an inverse association. Abbreviations: ALT, alanine aminotransferase; AST, aspartate aminotransferase; BMI, body mass index; GGT, gamma-glutamyl transferase; HDL, high-density lipoprotein cholesterol; LDL, low-density lipoprotein cholesterol; TG, triglycerides.

Correlation analyses using the 16-point scalp SD score as the primary severity measure showed that the strongest association was observed for GGT (rho = 0.745, 95% CI: 0.535 to 0.868, *p* < 0.001), followed by triglycerides (rho = 0.634, 95% CI: 0.366 to 0.805, *p* < 0.001), ALT (rho = 0.572, 95% CI: 0.285 to 0.765, *p* < 0.001), and the TG/HDL-C ratio (rho = 0.569, 95% CI: 0.275 to 0.766, *p* < 0.001) (Table 2, Figure 2). These prespecified primary associations remained statistically significant after false-discovery-rate adjustment.

Among exploratory variables, statistically significant positive correlations with the 16-point score were observed for non-HDL cholesterol (rho = 0.418, 95% CI: 0.081 to 0.669, *p* = 0.017), total cholesterol (rho = 0.397, 95% CI: 0.056 to 0.655, *p* = 0.024), and AST (rho = 0.361, 95% CI: 0.020 to 0.627, *p* = 0.039). After false-discovery-rate adjustment, the associations with non-HDL cholesterol and total cholesterol remained statistically significant, whereas the association with AST did not. LDL cholesterol showed a borderline positive association with the 16-point score (rho = 0.348, 95% CI: −0.001 to 0.621, *p* = 0.051), whereas HDL cholesterol showed a borderline inverse association (rho = −0.327, 95% CI: −0.607 to 0.024, *p* = 0.067). No significant correlations were observed for BMI or glucose.

The ranked correlation pattern is shown in Figure 2. Overall, the most consistent associations with scalp SD severity involved the prespecified triglyceride-related parameters and liver-associated enzymes.

### 3.4. Secondary Analysis Using the 0–36 Physician-Assessed Severity Score

When the study-specific 0–36 physician-assessed scalp SD score was analysed as a secondary, more granular measure of disease activity, the overall correlation pattern was reproduced and generally strengthened (Table 2). The strongest correlations were observed for triglycerides (rho = 0.834, 95% CI: 0.684 to 0.916, *p* < 0.001), GGT (rho = 0.822, 95% CI: 0.663 to 0.910, *p* < 0.001), the TG/HDL-C ratio (rho = 0.756, 95% CI: 0.553 to 0.874, *p* < 0.001), and ALT (rho = 0.692, 95% CI: 0.457 to 0.837, *p* < 0.001). These associations remained statistically significant after false-discovery-rate adjustment.

In this secondary analysis, total cholesterol, non-HDL cholesterol, LDL cholesterol, AST and BMI also correlated positively with disease severity, whereas HDL cholesterol correlated inversely. These exploratory associations remained statistically significant after false-discovery-rate adjustment. Glucose was not associated with either severity measure.

### 3.5. Adjusted and Sensitivity Analyses

To address potential confounding by age and BMI, exploratory rank-based partial correlation analyses were performed for the prespecified primary biochemical variables. After adjustment for age and BMI, the associations between the 16-point scalp SD severity score and the main biochemical parameters remained statistically significant: triglycerides (partial rho = 0.582, *p* < 0.001), TG/HDL-C ratio (partial rho = 0.494, *p* = 0.006), ALT (partial rho = 0.574, *p* < 0.001) and GGT (partial rho = 0.752, *p* < 0.001) (Supplementary Table S1).

Sensitivity analyses showed that the main correlation pattern was not driven by a single patient with high liver enzyme values. After exclusion of this patient, correlations with the 16-point scalp SD score remained significant for triglycerides (rho = 0.674, *p* < 0.001), TG/HDL-C ratio (rho = 0.677, *p* < 0.001), ALT (rho = 0.531, *p* = 0.002) and GGT (rho = 0.720, *p* < 0.001). The associations also remained directionally consistent and statistically significant in additional exploratory sensitivity analyses excluding patients receiving lipid-lowering therapy, patients with documented problematic alcohol use, current smokers or patients reporting no or low physical activity (Supplementary Table S1).

## 4. Discussion

In this exploratory study, scalp seborrheic dermatitis severity was associated with a distinct metabolic and liver-associated biochemical profile. The strongest associations with the 16-point scalp SD severity score were observed for GGT, triglycerides, ALT and the TG/HDL ratio. Importantly, the same pattern was reproduced and generally strengthened when the study-specific 0–36 physician-assessed score was used as a secondary, more granular measure of objective scalp disease activity. Moreover, the main associations remained present after exclusion of the patient with high liver enzyme elevations, suggesting that the observed pattern was not driven solely by a single outlier.

These findings fit well into the evolving concept of SD as a disorder of the lipid-rich cutaneous microenvironment rather than a purely superficial fungal disease. The classical pathogenic model of SD involves the interaction between *Malassezia* spp., sebaceous lipids and individual host susceptibility [4,5,6]. *Malassezia* yeasts depend on exogenous lipids and can hydrolyse sebaceous triglycerides, generating free fatty acids and lipid-derived metabolites that may induce irritation, inflammation and abnormal desquamation in susceptible individuals [5,6]. In this context, the scalp is not simply a passive anatomical site affected by SD, but a lipid-rich cutaneous microenvironment in which sebaceous gland activity, microbial metabolism, epidermal barrier integrity and host inflammatory responses converge.

Our results extend this local lipid model by suggesting that systemic lipid handling may also be related to clinical scalp disease severity. The most consistent lipid-related findings in our cohort involved triglycerides and the TG/HDL ratio, whereas associations with total cholesterol, LDL cholesterol and non-HDL cholesterol were weaker. This pattern may be clinically relevant because triglyceride-rich lipid metabolism is closely linked to broader metabolic dysfunction. Although our study was not designed to diagnose metabolic syndrome or insulin resistance, the association between scalp SD severity and triglyceride-related indices suggests that more severe disease may coexist with a systemic metabolic phenotype.

The biological plausibility of this association is supported by previous molecular studies of dandruff and scalp SD. Transcriptomic profiling has shown that lesional scalp skin is characterized by reciprocal activation of immune-response genes and downregulation of genes involved in lipid metabolism [7]. In parallel, structural and lipidomic studies have demonstrated impaired barrier function and alterations in stratum corneum lipid composition in SD lesions [8]. Therefore, the association between scalp SD severity and circulating lipid-related parameters observed in our study may reflect a broader disturbance of lipid homeostasis involving both the skin and systemic metabolism. This interpretation remains speculative, but it provides a coherent framework linking clinical severity, sebaceous biology, barrier impairment and metabolic profile.

Our findings are also consistent with earlier clinical studies suggesting an association between SD and metabolic abnormalities. Previous reports have linked SD with metabolic syndrome and its components, including dyslipidaemia, low HDL cholesterol and increased triglycerides [10,11,12]. Other studies have reported relationships between serum lipid parameters, sebum secretion and SD severity [13,14]. However, much of the existing literature has focused on the presence or absence of SD rather than on quantified scalp disease severity. The present study adds to this field by showing that severity scores themselves correlate with metabolic and liver-associated parameters, particularly triglycerides, TG/HDL ratio, ALT and GGT.

The association with liver-associated enzymes is perhaps the most original aspect of our study. GGT showed the strongest correlation with the primary 16-point severity score, and ALT was also consistently associated with disease severity. These findings should not be interpreted as evidence that liver dysfunction causes scalp SD. Rather, they suggest that severe scalp SD may coexist with a biochemical profile compatible with broader metabolic and liver-associated abnormalities. This is relevant because the liver plays a central role in triglyceride handling, lipoprotein metabolism and metabolic inflammation. Current concepts of metabolic dysfunction-associated steatotic liver disease emphasize the link between hepatic triglyceride accumulation and cardiometabolic risk factors [15]. Previous retrospective data also suggested that hepatic steatosis may be frequent among patients with SD, although disease severity was not assessed and liver-associated biochemical markers were not analysed in relation to cutaneous activity [16]. Our findings therefore provide a rationale for further studies investigating whether severe scalp SD may be associated with metabolic dysfunction-associated liver phenotypes.

The markedly higher frequency of *Malassezia* detection in patients with severe scalp SD supports the relevance of the fungal–lipid axis in disease activity. However, this finding should not be interpreted as evidence of a purely infectious mechanism. Contemporary reviews have emphasized that the role of *Malassezia* in SD is complex and that yeast density alone does not fully explain disease presence or severity [2,9]. Instead, *Malassezia* probably acts within a permissive host environment characterized by altered sebaceous lipid availability, barrier dysfunction and immune activation [2,4,9]. In our cohort, the coexistence of more frequent *Malassezia* detection with higher triglycerides, TG/HDL ratio, ALT and GGT supports a multidimensional model of severe scalp SD, in which microbial, cutaneous and systemic metabolic factors may interact.

Several observations argue against a simplistic explanation based only on age or obesity. Patients with severe scalp SD were younger than those with non-severe disease, whereas BMI was numerically higher but did not differ significantly between groups. In addition, selected comorbidities and medications potentially affecting lipid or liver-associated biochemical parameters were broadly comparable between severity groups. Lipid-lowering therapy, hypertension, thyroid hormone replacement and documented problematic alcohol use were infrequent and did not differ significantly between groups. Current smoking and low physical activity were more common among patients with severe scalp SD, although these differences did not reach statistical significance. These variables remain important potential confounders, but the available data suggest that the associations with triglycerides, TG/HDL ratio, ALT and GGT cannot be reduced solely to older age, overt obesity or a clear imbalance in major recorded comorbidities.

From a clinical perspective, these findings do not justify routine metabolic or hepatological screening in every patient with mild or typical SD. However, they suggest that severe, persistent, recurrent or treatment-refractory scalp SD may represent an opportunity to consider broader metabolic assessment, particularly when other cardiometabolic risk factors are present. In such patients, evaluation of lipid profile and liver-associated enzymes may be clinically reasonable, especially in the presence of overweight, central obesity, hypertension, diabetes, dyslipidaemia, low physical activity or suspected metabolic dysfunction. This approach should not be interpreted as disease-specific screening for liver disease in SD, but rather as a pragmatic reminder that severe scalp inflammation may coexist with systemic metabolic abnormalities that are clinically relevant beyond dermatology. Importantly, the present study does not define diagnostic thresholds or prove that treatment of lipid or liver-associated abnormalities improves scalp SD; these questions require prospective interventional studies.

The generalizability of these findings should be considered carefully. Because the study was conducted in a regional referral dermatology centre and included consecutive first-time patients presenting to this setting, the cohort may overrepresent individuals with persistent, diagnostically challenging or more severe scalp disease. Therefore, the observed associations may be most applicable to referral or specialist dermatology populations rather than to patients with mild, intermittent or self-managed SD in primary-care settings. Validation in larger, unselected and preferably multicentre cohorts is needed before these findings can be translated into broader clinical recommendations.

The main strength of this study is the use of two complementary severity measures. The published 16-point scalp SD scale was used as the primary measure, providing an externally defined clinical framework [18]. In parallel, the study-specific 0–36 physician-assessed score allowed a more detailed assessment of objective scalp activity across four scalp quadrants. The consistency of findings across both instruments strengthens the credibility of the observed associations. Additional strengths include the analysis of several biologically coherent lipid and liver-associated parameters, the recording of potentially relevant comorbidities, lifestyle-related factors and medication use, and the sensitivity analysis excluding the patient with extreme liver enzyme elevations.

This study also has important limitations. First, the sample size was small, with 34 patients included and complete primary 16-point severity data available in 33 patients. This limits statistical precision, increases the risk of unstable estimates and precludes robust multivariable modelling. Therefore, the results should be interpreted as exploratory and hypothesis-generating. Moreover, although consecutive first-time patients were screened during the study period, the study was conducted at a single regional referral dermatology centre. Patients attending such a centre may represent a more selected population, enriched for persistent, diagnostically challenging or more severe scalp disease, which may limit the generalizability of the findings to unselected primary-care or general dermatology populations.

Second, the observational cross-sectional design does not allow causal inference. It remains unclear whether metabolic and liver-associated abnormalities contribute to more severe scalp SD, whether severe SD is a marker of a broader inflammatory-metabolic phenotype, or whether both are consequences of shared upstream factors. Third, the study did not include a healthy control group, and therefore cannot determine whether the observed biochemical profile is specific to SD compared with unaffected individuals. Fourth, although selected potential confounders, including age, BMI, smoking, alcohol exposure, physical activity, comorbidities and lipid-lowering therapy, were recorded and explored in adjusted and sensitivity analyses, residual confounding cannot be excluded. In particular, the small sample size did not allow robust adjustment for multiple covariates, and the additional adjusted and sensitivity analyses should be interpreted as supportive rather than confirmatory.

Fifth, liver assessment was based on routine biochemical parameters; imaging-based assessment of hepatic steatosis, liver stiffness measurement, controlled attenuation parameter, insulin levels and HOMA-IR were not available. Sixth, alcohol exposure and physical activity were based on self-reported categorical data, without quantitative alcohol intake or objective activity monitoring. Seventh, *Malassezia* assessment was qualitative and performed as part of routine diagnostic work-up; quantitative fungal burden, species-level identification and broader microbiome profiling were not available. Finally, the 0–36 physician-assessed severity score was study-specific and not externally validated; formal inter-rater and intra-rater reliability testing was not performed. Therefore, this score should be interpreted as a secondary exploratory tool rather than a definitive severity instrument.

In conclusion, this exploratory study suggests that more severe scalp SD is associated with higher triglycerides, higher TG/HDL ratio and increased liver-associated enzymes, particularly GGT and ALT. These associations were observed using a published 16-point severity scale and were strengthened using a more granular physician-assessed 0–36 score. The findings support the concept that severe scalp SD may be linked to a broader lipid and liver-associated metabolic phenotype. Larger controlled studies incorporating standardized metabolic assessment, liver imaging, insulin-resistance markers and quantitative microbiome analysis are needed to validate these observations and to further investigate the proposed skin–lipid–liver axis in seborrheic dermatitis.

## Figures and Tables

**Table 1:** Patient characteristics according to scalp seborrheic dermatitis severity.

Parameter	Non-Severe Scalp SD (*n* = 15)	Severe Scalp SD (*n* = 18)	*p*-Value
Age, years	34.0 (21–83)	27.5 (19–61)	0.017
Female sex, *n* (%)	7/15 (46.7)	6/18 (33.3)	0.493
BMI, kg/m^2^	23.5 (19.7–30.0)	26.7 (18.6–38.6)	0.100
*Malassezia* detected, *n* (%)	3/15 (20.0)	16/18 (88.9)	<0.001
Lipid-lowering therapy, *n* (%)	1/15 (6.7)	3/18 (16.7)	0.607
Hypertension, *n* (%)	5/15 (33.3)	6/18 (33.3)	1.0
Type 2 diabetes, *n* (%)	0/15 (0.0)	1/18 (5.6)	1.0
Thyroid hormone replacement, *n* (%)	2/15 (13.3)	2/18 (11.1)	1.0
Urate-lowering therapy, *n* (%)	0/15 (0.0)	1/18 (5.6)	1.0
Current smoking, *n* (%)	3/15 (20.0)	7/18 (38.9)	0.283
Alcohol exposure			1.0
No current alcohol use, *n* (%)	3/15 (20.0)	3/18 (16.7)	
Occasional alcohol use, *n* (%)	8/15 (53.3)	10/18 (55.6)	
Documented problematic alcohol use, *n* (%)	1/15 (6.7)	2/18 (11.1)	
Alcohol data unavailable, *n* (%)	3/15 (20.0)	3/18 (16.7)	
Physical activity			0.140
Physical activity data unavailable, *n* (%)	2/15 (13.3)	3/18 (16.7)	
No/low physical activity, *n* (%)	3/15 (20.0)	9/18 (50.0)	
Occasional physical activity, *n* (%)	8/15 (53.3)	3/18 (16.7)	
Guideline-consistent physical activity, *n* (%)	2/15 (13.3)	3/18 (16.7)	
Triglycerides, mg/dL	78.0 (49.0–163.0)	116.0 (47.9–278.0)	0.004
TG/HDL ratio	1.2 (0.59–2.82)	2.6 (0.7–7.28)	0.006
ALT, U/L	20.0 (11.0–72.0)	46.0 (13.0–295.0)	0.028
GGT, U/L	11.0 (2.0–51.0)	33.0 (8.0–573.0)	0.003

Data are presented as median (min-max) or *n* (%), unless otherwise stated. Severe scalp seborrheic dermatitis was defined as a 16-point scalp seborrheic dermatitis score ≥ 10; non-severe disease was defined as a score < 10. Alcohol exposure and physical activity were recorded from structured medical history and are presented as categorical self-reported variables. ALT, alanine aminotransferase; BMI, body mass index; GGT, gamma-glutamyl transferase; HDL, high-density lipoprotein cholesterol; SD, seborrheic dermatitis; TG, triglycerides.

**Table 2:** Spearman correlations between scalp seborrheic dermatitis severity and metabolic or liver-associated parameters.

Parameter	16-Point Score Rho (95% CI)	*p*-Value	FDR-Adjusted *p*-Value	0–36 Score Rho (95% CI)	*p*-Value	FDR-Adjusted *p*-Value
BMI	0.278 (−0.072 to 0.567)	0.117	0.129	0.374 (0.035 to 0.636)	0.032	0.035
Glucose	−0.098 (−0.529 to 0.373)	0.689	0.689	−0.076 (−0.513 to 0.393)	0.758	0.758
Total cholesterol	0.397 (0.056 to 0.655)	0.024	0.045	0.636 (0.369 to 0.806)	<0.001	<0.001
HDL cholesterol	−0.327 (−0.607 to 0.024)	0.067	0.082	−0.420 (−0.671 to −0.084)	0.017	0.020
LDL cholesterol	0.348 (−0.001 to 0.621)	0.051	0.071	0.582 (0.293 to 0.774)	<0.001	<0.001
Non-HDL cholesterol	0.418 (0.081 to 0.669)	0.017	0.038	0.663 (0.409 to 0.822)	<0.001	<0.001
Triglycerides	0.634 (0.366 to 0.805)	<0.001	<0.001	0.834 (0.684 to 0.916)	<0.001	<0.001
TG/HDL ratio	0.569 (0.275 to 0.766)	<0.001	0.002	0.756 (0.553 to 0.874)	<0.001	<0.001
ALT	0.572 (0.285 to 0.765)	<0.001	0.002	0.692 (0.457 to 0.837)	<0.001	<0.001
AST	0.361 (0.02 to 0.627)	0.039	0.061	0.484 (0.169 to 0.709)	0.004	0.006
GGT	0.745 (0.535 to 0.868)	<0.001	<0.001	0.822 (0.663 to 0.910)	<0.001	<0.001

Data are presented as Spearman’s rank correlation coefficients (rho) with 95% confidence intervals, unadjusted *p*-values and false-discovery-rate-adjusted *p*-values calculated using the Benjamini–Hochberg method. The 16-point score refers to the published scalp seborrheic dermatitis severity scale; the 0–36 score refers to the study-specific physician-assessed scalp severity score. The prespecified primary biochemical variables were triglycerides, TG/HDL-C ratio, ALT and GGT. Other variables were considered exploratory. ALT, alanine aminotransferase; AST, aspartate aminotransferase; BMI, body mass index; CI, confidence interval; FDR, false discovery rate; GGT, gamma-glutamyl transferase; HDL, high-density lipoprotein cholesterol; LDL-C, low-density lipoprotein cholesterol; TG, triglycerides.

## Data Availability

All data generated or analyzed during this study are included in this article (and its Supplementary Material Files). Further enquiries can be directed to the corresponding author.

## Supplementary Materials

The following supporting information can be downloaded at: https://www.mdpi.com/article/10.3390/jcm15186937/s1, Table S1: Adjusted and sensitivity analyses for associations between the 16-point scalp seborrheic dermatitis severity score and prespecified biochemical variables. Figure S1: Study-specific 0–36 physician-assessed scalp seborrhoeic dermatitis severity score.

## References

[1] Dall’Oglio F., Nasca M.R., Gerbino C., Micali G. (2022). An overview of the diagnosis and management of seborrheic dermatitis. Clin. Cosmet. Investig. Dermatol..

[2] Adalsteinsson J.A., Kaushik S., Muzumdar S., Guttman-Yassky E., Ungar J. (2020). An update on the microbiology, immunology and genetics of seborrheic dermatitis. Exp. Dermatol..

[3] Sampogna F., Linder D., Piaserico S., Altomare G., Bortune M., Calzavara-Pinton P., Vedove C., Girolomoni G., Peserico A., Sala R. (2014). Quality of life assessment of patients with scalp dermatitis using the Italian version of the Scalpdex. Acta Derm. Venereol..

[4] Schwartz J.R., Messenger A.G., Tosti A., Todd G., Hordinsky M., Hay R.J., Wang X., Zachariae C., Kerr K., Henry J. (2013). A comprehensive pathophysiology of dandruff and seborrheic dermatitis—Towards a more precise definition of scalp health. Acta Derm. Venereol..

[5] Ro B.I., Dawson T.L. (2005). The role of sebaceous gland activity and scalp microfloral metabolism in the etiology of seborrheic dermatitis and dandruff. J. Investig. Dermatol. Symp. Proc..

[6] De Angelis Y.M., Gemmer C.M., Kaczvinsky J.R., Kenneally D.C., Schwartz J.R., Dawson T.L. (2005). Three etiologic facets of dandruff and seborrheic dermatitis: Malassezia fungi, sebaceous lipids, and individual sensitivity. J. Investig. Dermatol. Symp. Proc..

[7] Mills K.J., Hu P., Henry J., Tamura M., Tiesman J.P., Xu J. (2012). Dandruff/seborrhoeic dermatitis is characterized by an inflammatory genomic signature and possible immune dysfunction: Transcriptional analysis of the condition and treatment effects of zinc pyrithione. Br. J. Dermatol..

[8] Rousel J., Nădăban A., Saghari M., Pagan L., Zhuparris A., Theelen B., Gambrah T., van der Wall H.E.C., Vreeken R.J., Feiss G.L. (2024). Lesional skin of seborrheic dermatitis patients is characterized by skin barrier dysfunction and correlating alterations in the stratum corneum ceramide composition. Exp. Dermatol..

[9] Chang C.H., Chovatiya R. (2024). More yeast, more problems? Reevaluating the role of Malassezia in seborrheic dermatitis. Arch. Dermatol. Res..

[10] Woolhiser E., Keime N., Patel A., Weber I., Adelman M., Dellavalle R.P. (2024). Nutrition, obesity, and seborrheic dermatitis: Systematic review. JMIR Dermatol..

[11] Imamoglu B., Hayta S.B., Guner R., Akyol M., Ozcelik S. (2016). Metabolic syndrome may be an important comorbidity in patients with seborrheic dermatitis. Arch. Med. Sci. Atheroscler. Dis..

[12] Akbaş A., Kılınç F., Şener S., Hayran Y. (2022). Investigation of the relationship between seborrheic dermatitis and metabolic syndrome parameters. J. Cosmet. Dermatol..

[13] Toruan T.L., Nopriyati Theodorus Sari Y.M. (2017). The relationship between serum lipid profile and sebum secretion in seborrheic dermatitis patients. Int. J. Health Sci. Res..

[14] Abdulrahman B.B., Elethawi A.M.D., Abdullah H.M. (2020). Assessment of lipid profile among patients with seborrheic dermatitis. Bali Med. J..

[15] European Association for the Study of the Liver, European Association for the Study of Diabetes, European Association for the Study of Obesity (2024). EASL–EASD–EASO Clinical Practice Guidelines on the management of metabolic dysfunction-associated steatotic liver disease (MASLD). J. Hepatol..

[16] Aktas H., Oner S., Cebecik A. (2019). A retrospective analysis on the concomitance of seborrheic dermatitis and fatty liver disease. Med. Sci..

[17] Clark G.W., Pope S.M., Jaboori K.A. (2015). Diagnosis and treatment of seborrheic dermatitis. Am. Fam. Physician.

[18] Zhang F., Li Y., Ren W., Li S. (2023). Establishment of clinical evaluation criteria for scalp seborrheic dermatitis. J. Cosmet. Dermatol..

