# Supplementary material for: Scalp Seborrheic Dermatitis Severity Is Associated with Lipid and Liver-Associated Biochemical Parameters
Source: J Clin Med. 2026 Sep 8;15(18):6937. doi: 10.3390/jcm15186937 (PMC13607883; doi:10.3390/jcm15186937)
Supplement: Supplementary file 1 [file jcm-15-06937-s001.zip › jcm-4531225-supplementary.pdf]

Supplementary Figure S1. Study-specific 0–36 physician-assessed scalp seborrhoeic dermatitis severity score

The score was calculated in four scalp quadrants. In each quadrant, erythema, scaling/flaking and Wood's lamp fluorescence were assessed. The total score is the sum of the four quadrant scores and ranges from 0 to 36 points. Higher scores indicate more severe scalp disease.

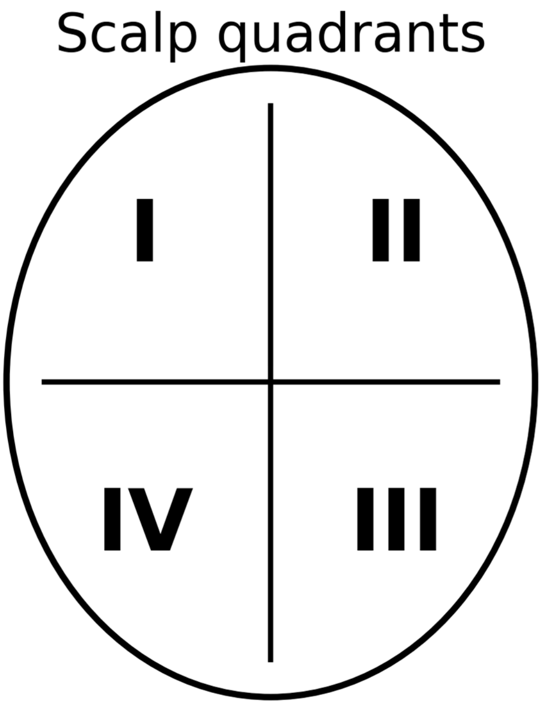

Scalp quadrant scheme used for the 0–36 severity assessment.

Scoring domains

Domain	Score	Definition
Erythema	0–4	0 = absent; 1 = mild; 2 = moderate; 3 = severe; 4 = very severe.
Scaling/flaking	0–4	0 = absent; 1 = mild; 2 = moderate; 3 = severe; 4 = very severe.
Wood's lamp fluorescence	0–1	0 = absent; 1 = present.
Quadrant score	0–9	Sum of erythema, scaling/flaking and Wood's lamp fluorescence in one scalp quadrant.
Total score	0–36	Sum of quadrant I, II, III and IV scores.

Formula: total 0–36 score = (quadrant I score + quadrant II score + quadrant III score + quadrant IV score).

Note: The 0–36 score was used as a study-specific secondary physician-assessed measure of objective scalp disease activity. Pruritus was recorded separately using a 0–10 numerical rating scale.

Supplementary Table S1. Adjusted and sensitivity analyses for associations between the 16-point scalp seborrheic dermatitis severity score and prespecified biochemical variables

Analysis	Variable	n	rho	p-value
Unadjusted primary analysis	Triglycerides	32	0.634	<0.001
Unadjusted primary analysis	TG/HDL-C ratio	32	0.569	<0.001
Unadjusted primary analysis	ALT	33	0.572	<0.001
Unadjusted primary analysis	GGT	32	0.745	<0.001

Partial correlation adjusted for age and BMI	Triglycerides	32	0.582	<0.001
Partial correlation adjusted for age and BMI	TG/HDL-C ratio	32	0.494	0.006
Partial correlation adjusted for age and BMI	ALT	33	0.574	<0.001
Partial correlation adjusted for age and BMI	GGT	32	0.752	<0.001
Excluding the patient with high liver enzyme values	Triglycerides	31	0.674	<0.001
Excluding the patient with high liver enzyme values	TG/HDL-C ratio	31	0.677	<0.001
Excluding the patient with high liver enzyme values	ALT	32	0.531	0.002
Excluding the patient with high liver enzyme values	GGT	31	0.720	<0.001
Excluding patients receiving lipid-lowering therapy	Triglycerides	28	0.611	<0.001
Excluding patients receiving lipid-lowering therapy	TG/HDL-C ratio	28	0.513	0.005
Excluding patients receiving lipid-lowering therapy	ALT	29	0.516	0.004
Excluding patients receiving lipid-lowering therapy	GGT	28	0.730	<0.001
Excluding patients with documented problematic alcohol use	Triglycerides	29	0.613	<0.001
Excluding patients with documented problematic alcohol use	TG/HDL-C ratio	29	0.542	0.002
Excluding patients with documented problematic alcohol use	ALT	30	0.528	0.003
Excluding patients with documented problematic alcohol use	GGT	29	0.735	<0.001
Excluding current smokers	Triglycerides	23	0.739	<0.001
Excluding current smokers	TG/HDL-C ratio	23	0.683	<0.001
Excluding current smokers	ALT	23	0.419	0.047
Excluding current smokers	GGT	23	0.664	<0.001
Excluding patients reporting no/low physical activity	Triglycerides	20	0.565	0.01
Excluding patients reporting no/low physical activity	TG/HDL-C ratio	20	0.478	0.03
Excluding patients reporting no/low physical activity	ALT	21	0.529	0.014
Excluding patients reporting no/low physical activity	GGT	20	0.728	<0.001

Data are presented as correlation coefficients with corresponding p-values. For the unadjusted primary analysis and sensitivity analyses, rho denotes Spearman's rank correlation coefficient. For partial correlation analyses, rho denotes rank-based partial Spearman's correlation coefficient adjusted for age and BMI. All analyses refer to associations with the 16-point scalp seborrheic dermatitis severity score. Sensitivity analyses were performed for the prespecified primary biochemical variables. ALT, alanine aminotransferase; BMI, body mass index; GGT, gamma-glutamyl transferase; HDL-C, high-density lipoprotein cholesterol; TG, triglycerides.
